# Administration of S-nitrosoglutathione after traumatic brain injury protects the neurovascular unit and reduces secondary injury in a rat model of controlled cortical impact

**DOI:** 10.1186/1742-2094-6-32

**Published:** 2009-11-04

**Authors:** Mushfiquddin Khan, Yeong-Bin Im, Anandakumar Shunmugavel, Anne G Gilg, Ramanpreet K Dhindsa, Avtar K Singh, Inderjit Singh

**Affiliations:** 1 Department of Pediatrics, Medical University of South Carolina, Charleston, SC 29425, USA; 2 Department of Pathology and Laboratory Medicine, Medical University of South Carolina, Charleston, SC 29425, USA; 3 Ralph H. Johnson V. A. Medical Center, Charleston, SC 29401, USA

## Abstract

**Background:**

Traumatic brain injury (TBI) is a major cause of preventable death and serious morbidity in young adults. This complex pathological condition is characterized by significant blood brain barrier (BBB) leakage that stems from cerebral ischemia, inflammation, and redox imbalances in the traumatic penumbra of the injured brain. Once trauma has occurred, combating these exacerbations is the keystone of an effective TBI therapy. Following other brain injuries, nitric oxide modulators such as S-nitrosoglutathione (GSNO) maintain not only redox balance but also inhibit the mechanisms of secondary injury. Therefore, we tested whether GSNO shows efficacy in a rat model of experimental TBI.

**Methods:**

TBI was induced by controlled cortical impact (CCI) in adult male rats. GSNO (50 μg/kg body weight) was administered at two hours after CCI. GSNO-treated injured animals (CCI+GSNO group) were compared with vehicle-treated injured animals (CCI+VEH group) in terms of tissue morphology, BBB leakage, edema, inflammation, cell death, and neurological deficit.

**Results:**

Treatment of the TBI animals with GSNO reduced BBB disruption as evidenced by decreased Evan's blue extravasation across brain, infiltration/activation of macrophages (ED1 positive cells), and reduced expression of ICAM-1 and MMP-9. The GSNO treatment also restored CCI-mediated reduced expression of BBB integrity proteins ZO-1 and occludin. GSNO-mediated improvements in tissue histology shown by reduction of lesion size and decreased loss of both myelin (measured by LFB staining) and neurons (assayed by TUNEL) further support the efficacy of GSNO therapy. GSNO-mediated reduced expression of iNOS in macrophages as well as decreased neuronal cell death may be responsible for the histological improvement and reduced exacerbations. In addition to these biochemical and histological improvements, GSNO-treated injured animals recovered neurobehavioral functions as evaluated by the rotarod task and neurological score measurements.

**Conclusion:**

GSNO is a promising candidate to be evaluated in humans after brain trauma because it not only protects the traumatic penumbra from secondary injury and improves overall tissue structure but also maintains the integrity of BBB and reduces neurologic deficits following CCI in a rat model of experimental TBI.

## Background

Immediately following traumatic brain injury (TBI), the direct trauma and lack of cerebral blood flow (CBF) to injured tissue cause necrotic neuronal cell death. However, even greater apoptotic neuronal loss occurs hours and days later as a result of secondary injury from cerebral ischemia/hypoxia, blood brain barrier (BBB) leakage, and inflammatory/oxidative stress [1]. In this respect, TBI has similar pathophysiology to ischemic stroke. Both have hypoxia, disrupted BBB, edema, inflammation, neurodegeneration, and neurological deficits [2,3]. Furthermore, the time-dependant progression of the disease presents a window of opportunity to reduce secondary injury-mediated damage in both stroke and TBI. Regional ischemia early after TBI and a relationship between the volume of ischemic tissue and neurological outcomes have been reported [2].

However, major clinical trials with drugs that target only neuroprotection following TBI have proven to be of little efficacy [4]. TBI is a complex disease involving multiple tissues such as vasculature, vascular immune cells, and various cell types within the central nervous system (CNS). The failure of therapies targeted only to neuronal protection is attributable, in part, to the lack of concomitant protection of cerebral blood vessels from the secondary injury of inflammation and reactive oxygen species/reactive nitrogen species (ROS/RNS) stress. Recently, we have documented that S-nitrosoglutathione (GSNO), a nitric oxide (NO) modulator and endogenous nitrosylating agent, not only protects endothelial function from oxidative and inflammatory injuries [5] but also lessens ischemia reperfusion (IR) injury in a rat model of experimental stroke [6,7]. We hypothesize a similar efficacy of GSNO in ameliorating the secondary injury components of TBI. Therefore, we tested its therapeutic potential in a rat model of controlled cortical impact (CCI).

GSNO is a physiological metabolite of glutathione (GSH) and NO [8,9]. It is involved in several pharmacological activities [10] and cellular signaling [11-15]. GSNO is present in micromolar concentrations in the rat brain [16]. It is several-fold more potent than GSH against oxidative stress caused by ONOO^- ^[17]. Unlike other classes of NO donor, GSNO is a stable compound and does not decompose spontaneously; it requires additional agents or enzymes, including GSNO reductase or the thioredoxin system [18,19], for its metabolism. It reduces the frequency of embolic signals [20-22] and can reverse acute vasoconstriction, preventing ischemic brain injury after subarachnoid hemorrhage [23]. Administration of GSNO has been shown to suppress inducible nitric oxide synthase (iNOS) induction and enhance endothelial nitric oxide synthase (eNOS) expression in pedicle vessels, resulting in blood perfusion and a higher flap survival after I/R [24,25]. In animals, GSNO treatment blocks inflammation and secondary injury in the brain following IR [6,7]. The deleterious role of inflammation in TBI is also reported to be mediated via induction of cytokines, chemokines and iNOS [26]. Expression of iNOS has been shown at 3 to 7 days after TBI in adult rats, predominantly in macrophages, and its pharmacological inhibition by aminoguanidine reduces TBI [27], supporting the concept that down regulation of iNOS in TBI is beneficial. The antiinflammatory activity of GSNO in down regulating iNOS is mediated by the inhibition of NF-κB activation in rat primary astrocytes and microglial cell lines [7].

Injuries that compromise the integrity of the BBB lead to the activation of endothelial cells and the recruitment of inflammatory cells in the CNS. Increased expression of ICAM-1 and MMP-9 has been reported in TBI, and the ICAM-1 antibody is reported to improve recovery following TBI [28]. MMP-9 can degrade crucial components of BBB and exacerbate edema. Moreover, its expression is associated with inflammation [29]. Following TBI, BBB disruption is a common clinical condition which leads to an increase in intracranial pressure caused by severe brain edema. Brain edema is formed due to cellular injury and circulatory collapse [30]. The microvascular permeability increases due, in part, to secondary oxidative stress [31,32], which is related to endothelial cell activation and endothelial dysfunction. Suppression of neuroinflammation and edema improves functional and neurologic outcomes [26].

Neurobehavioral deficits, especially in motor and cognitive functions, are often the cause of significant disability and morbidity after TBI. Motor impairments after moderate-to-severe TBI play a significant role in the initial recovery phase [33]. Determining the extent of clinically relevant neuropathology (defined as neuropathology associated with persistent neurobehavioral deficits) associated with TBI, particularly in the milder spectrum, is problematic. Nevertheless, evaluation of improvement in neurological function using a clinically relevant TBI model such as controlled cortical impact (CCI) establishes the efficacy and the clinical relevance of an experimental therapy [34].

The results in this study document that treatment of the injured rats at two hours after CCI with GSNO reduced BBB leakage and improved neurobehavioral functions. The treatment also blocked infiltration of macrophages and reduced the expression of ICAM-1, MMP-9, and iNOS. GSNO also inhibited the TBI-mediated decrease in the expression of ZO-1 and occludin. Improvement in tissue histology and decreased loss of both myelin and neurons in the GSNO-treated injured group further support the efficacy of the GSNO therapy.

## Methods

### Reagents

GSNO was purchased from World Precision Instruments (Sarasota, FL). All other chemicals and reagents used were purchased from Sigma (St. Louis, MO), unless stated otherwise.

### Animals

Male Sprague-Dawley rats weighing 240-260 g (Harlan Laboratories, Wilmington, MA) were used in this study. All animal procedures were approved by the Medical University of South Carolina Animal Review Committee and received humane care in compliance with the Medical University of South Carolina's experimental guidelines and the National Research Council's criteria for humane care (*Guide for the Care and Use of Laboratory Animals*).

### Experimental design and administration of GSNO

The rats were randomly allocated into the three groups: i) Sham (sham-operated control, n = 29), ii) CCI+VEH (vehicle-treated CCI, n = 29), iii) CCI+GSNO (GSNO-treated CCI, n = 29). Because GSNO-treated sham animals (n = 5) had no altered physiologic parameters including blood pressure (BP) and heart rate (HR) (data not shown), we did not include the Sham+GSNO group for further study. In the CCI+GSNO group, the rats were given GSNO (50 μg/kg body weight), dissolved in sterile PBS (~300 μl) by mouth using gavage needle at 2 hours after CCI. The rats in the CCI+VEH and the Sham groups were administered the same volume of PBS. The same dose of GSNO was repeated every 24 hours by mouth until the end point as described under each experiment. The dose used in this study was determined by a dose response curve ranging from 10 μg to 100 μg/kg body weight. The dose 50 μg/kg was found most effective in reducing contusion volume measured at 7 days after CCI. The selected dose did not alter physiologic parameters, including BP, HR, and temperature, measured at 1 hour following GSNO treatment. Similar observations with low dose GSNO treatments were made in the experimental stroke study [6,7]. Therefore, we did not include data from physiologic measurements in this study.

### Controlled cortical impact (CCI) model of TBI

Surgical anesthesia was induced by ketamine (90 mg/kg body weight) and xylazine (10 mg/kg body weight) administered intraperitoneally (ip). Analgesic buprenorphine was administered pre-emptively to alleviate the pain following surgery. Following endotracheal intubation, the animals were secured in a stereotaxic frame and ventilated mechanically. A dab of sterile ophthalmic ointment was placed on each eye to compensate for the decrease in lacrimation during anesthesia. Utilizing aseptic techniques, a midline scalp incision was made, and the skin and fascia were reflected to expose the skull. A craniotomy was made in the right hemisphere encompassing bregma and lambda and between the sagittal suture and the coronal ridge with a handheld Michele trephine. The resulting bone flap was removed, and the craniotomy enlarged further with cranial rongeurs. CCI injury was produced as previously described in the extensive literature [34-39]. A cortical contusion was produced on the exposed cortex using a controlled impactor device TBI-0310 TBI Model system (Precision Systems and Instrumentation, LLC, Fairfax Station, VA). Briefly, the impacting shaft was extended, and the impact tip was centered and lowered over the craniotomy site until it touched the dura mater. Then, the rod was retracted and the impact tip was advanced farther to produce a brain injury of moderate severity for rats (tip diameter, 4 mm; cortical contusion depth, 3 mm; impact velocity, 1.5 m/sec) [37]. The impact tip was wiped clean with sterile alcohol after each impact and cleaned/disinfected further with cidex after surgery. Core temperature was maintained at 37 ± 0.5°C with a heating pad during surgery and recorded with a rectal probe. Immediately after injury, the skin incision was closed with nylon sutures, and 2% lidocaine jelly was applied to the lesion site to minimize any possible discomfort.

### Histology

The paraffin embedded brain was sectioned at a thickness of 4 μM using a Leica 2135 microtome (Deerfield, IL, USA). Brain sections following CCI were evaluated for tissue histology by morphometric analysis using Hematoxylin and Eosin (H&E) and Luxol Fast Blue (LFB)-PAS staining as described earlier [40].

### Lesion size measurement

Seven days after CCI, brains were removed, sectioned and stained with H&E. The images of the stained specimens were captured by a digital photo camera and analyzed by Image Pro for morphometric measurement. The total lesion volume was determined by integrating the volumes at each coronal section interval as reported [41,42]. A blind investigator performed the lesion volume analyses.

### Evaluation of BBB disruption by Evan's blue (EB) extravasation

BBB leakage was assessed by the method of Weismann and Stewart [43] with slight modification. The rats received 100 μl of a 5% solution of EB in saline administered intravenously at 4 hours after CCI. At 48 hours, cardiac perfusion was performed under deep anesthesia with 200 ml of saline to clear the cerebral circulation of EB. The brain was removed, sliced, and photographed. The two hemispheres were isolated and mechanically homogenized in 750 μl of N, N-dimethylformamide (DMF). The suspension obtained was kept at room temperature in the dark for 72 hr. It was centrifuged at 10,000 × g for 25 minutes, and the supernatant was spectrofluorimetrically analyzed (λ_ex _620 nm, λ_em _680 nm).

### Measurement of Edema (brain water content)

At 24 h following CCI, animals were euthanized to determine brain water content (edema). The cortices, excluding the cerebellum, were quickly removed, and the contralateral and ipsilateral hemispheres separately weighed. Each hemisphere was dried at 60°C for 72 hours, and the dry weight was determined. Water content was calculated in ipsilateral hemisphere as: water content (%) = (wet weight - dry weight)/wet weight × 100.

### Immunohistochemistry

Paraffin-embedded sections from the formalin-fixed brain tissues were stained for ED1, ICAM-1, and MMP-9. In brief, the brain tissue sections were deparaffinized, sequentially rehydrated in graded alcohol, and then immersed in PBS (pH 7.4). Slides were then microwaved for 2 mins in antigen unmasking solution (Vector Labs), cooled, and washed 3 times for 2 mins in PBS. Sections were incubated overnight with antibodies of ED1 (Accurate, Westbury, NY, USA), ICAM-1 (BD Pharmingen, San Jose, CA, USA), MMP-9, and iNOS (Santa Cruz Biotechnology Inc, Santa Cruz, CA, USA). They were then rinsed 3 times for 5 mins in PBS containing 0.1% Tween-20. Secondary anti- anti-mouse IgG conjugated with Alexa Fluor 488 was incubated on slides for 30 mins. Slides were examined for immunofluorescence in the traumatic penumbra area using an Olympus microscope equipped for epifluorescence and DP Controller software. Figures were compiled in Adobe Photoshop 7.0.

For double labeling, section was incubated first with antibody of iNOS followed by specific cell marker antibodies (GFAP, ED1, CD34, or NSE). For immunofluorescent double-labeling, immune complexes were visualized with Texas Red conjugated anti-rabbit IgG (1:100, Vector Labs) and FITC conjugated anti-mouse IgG (1:100, Vector Labs). Rabbit polyclonal IgG was used as a control primary antibody. Section was also incubated with FITC or Texas Red conjugated IgG without the primary antibody as a negative control. Slides were examined for immunofluorescence using an Olympus microscope equipped for epifluorescence filter and Adobe Photoshop software [7].

Cell counting was performed in three equi-distant sections (10 sections apart) from each brain and expressed as an average number of immunopositive cells per section. The positive cells were counted on each section at 400 × magnification (one visual field = 0.69 mm^2^) using an Olympus microscope equipped for epifluorescence and DP Controller software. Images were captured and processed using Adobe Photoshop 7.0.

### Fluorescent TUNEL assay for detection of apoptosis

Terminal deoxynucleotidyl transferase-mediated biotinylated UTPnick end labeling (TUNEL) assay was performed in the traumatic penumbra region by using an Apoptagfluorescein in situ apoptosis detection kit (Serological Corporation, Norcross, GA, USA) according to the manufacturer's protocol. For double labeling, brain sections from three animals in each experimental group were probed with mouse NSE (Chemicon-Millipore Billerica, MA, USA) and visualized by fluorescence microscopy as described previously [7]. TUNEL/NSE double positive cells were counted in at least ten microscopic frames of the sections and in equivalent areas of sections from animals in GSNO group.

### Western blot analysis

Western blot was performed in the traumatic penumbra area from the ipsilateral injured brain and a similar area from the control and/or contralateral tissues using antibodies ZO-1, occludin (Invitrogen Corporation, Carlsbad, CA, USA), and β-actin, as described earlier [7,44]. Protein concentrations were determined using protein assay dye from Bio-Rad Laboratories (Hercules, CA, USA).

### Neurological functional evaluation

Neurological motor measurement was performed using the Modified Neurological Severity Score (mNSS) as described [45]. These tests were performed on all rats 1 day before and after 6 days of CCI. All measurements were performed by observers blinded to treatment. The test is sensitive to unilateral cortical injury because it reflects multiple asymmetries, including postural, sensory, and forelimb and hind limb use asymmetries. A detailed description of this functional test has been previously reported [46]. Briefly, this motor test (total score points 18) is based on 1) raising the rat by tail and recording flexion (3 points), 2) walking on the floor (3 points), 3) sensory test (2 points), 4) beam balance tests (6 points), and reflexes absence and abnormal movements (4 points). Point is awarded for the inability to perform the tasks or for the lack of a tested reflex. Levels of injury in animals are determined as severe (13-18 points), moderate (7-12 points) and mild (1-6 points).

### Sensorimotor deficit behavioral test

Sensorimotor deficit was assessed by investigators blinded to the study conditions by the adhesive tape-removal test before CCI and 7, 14, and 21 days after. Two pieces of adhesive-backed paper dots (113.1 mm^2^) were used as bilateral tactile stimuli attached at the distal-radial region on each forelimb. The latency was recorded (the time to remove each stimulus from the forelimbs) during three trials per day for each forepaw as described [47]. Before injury, rats were trained for 3 days. The test has been validated in a CCI rat model of TBI [48].

### Rotarod

Latency on rotarod was measured using the Rotarod system (Rotomex 5) from Columbus Instruments, Columbus, OH, by trained personnel blinded to animal groups as described [49-52]. Animals were pre-trained on an automated 4-lane rotarod unit and the task was performed up to two weeks after CCI. The animal was placed on the rod and tested for its latency period. The starting speed was set to 0, and the speed was increased by 2 rpm every 5 seconds up to 30 rpm. Total time in seconds that the animal could stay on the drum was recorded. Each animal was given 3 trials, and the mean latency of three trials was calculated for each animal.

### Statistical evaluation

Statistical analysis was performed as described [53] using software Graphpad Prism 3.0. Unless otherwise stated, all the values were expressed as mean ± SD of n determinations or as mentioned. The results from biochemical and animal behavior studies were examined by unpaired *t*-test. Multiple comparisons were performed using Kruskal-Wallis test or using ANOVA followed by Bonferroni test as appropriate. A p value less than 0.05 was considered significant.

## Results

### GSNO is protective in CCI model of TBI and reduces expression of ED1 and ICAM-1

**Figure 1:**
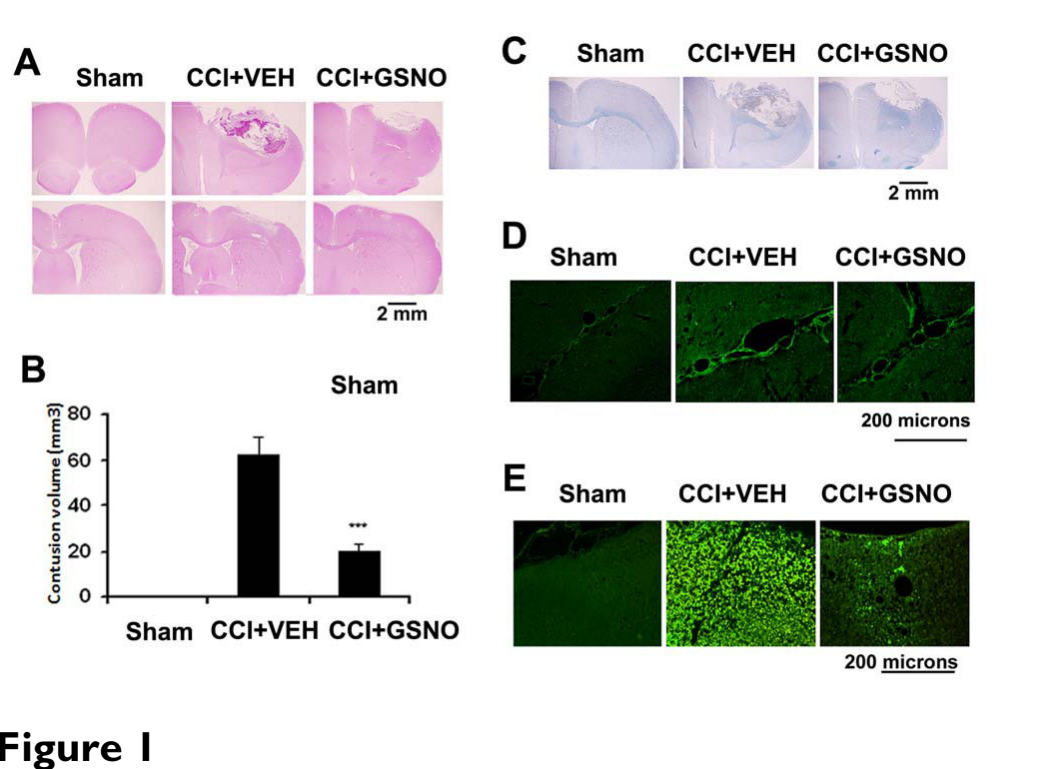
**Effect of GSNO treatment on TBI-mediated tissue injury and on the expression of ICAM-1 and ED1 in ipsilateral rat brain after CCI**. Animals were treated with GSNO (0.05 mg/kg) 2 h following CCI. Seven days after TBI, brains were removed, sectioned and stained with H&E (A) and LFB (C) and analyzed for lesion size (B) using morphometric measurement. GSNO reduced TBI-mediated tissue injury, decreased lesion size (B), and reduced the expression of ICAM-1 (D) and ED1 (E) (detected in traumatic penumbral area at 72 h following TBI). Photomicrographs are the representative of n = 3 in each group. Lesion size data are presented as mean ± SD (n = 3). ***p < 0.001 vs. CCI+VEH.

We have previously documented GSNO not only as a potent neuroprotective agent but also as an endothelium protecting agent in a rat model of experimental stroke. Therefore, we investigated whether the IR injury-reducing agent GSNO also reduces neurovascular injury when administered in a clinically relevant 2 hours post-injury rat CCI model of TBI. Animals were administered GSNO (0.05 mg/kg, orally) or PBS (VEH) at 2 h following CCI. The same dose was repeated every 24 h by mouth until the animals were sacrificed. Brain sections at day 7 following CCI were evaluated for tissue histology by morphometric analysis using Hematoxylin and Eosin (H&E) (Fig 1A) and (Luxol Fast Blue (LFB)-PAS staining (Fig 1C). Contusion size analysis was performed using H&E sections (Fig 1B).

GSNO treatment significantly improved tissue structure/morphology and reduced contusion size (62.3 ± 7.9 vs. 20.4 ± 2.8 GSNO; p < 0.001) (Fig 1A-B), supporting the potential of GSNO therapy in TBI. The treatment with GSNO also improved LFB staining (Fig 1C), indicating further that GSNO protected against the loss of myelin following TBI. Furthermore, GSNO decreased the expression of ICAM-1 (Fig 1D) (a mediator of endothelial cell activation leading to BBB disruption) and ED-1 (Fig 1E) (a marker of activated macrophages and microglia) determined at 72 h after CCI. Enhanced expression of ICAM-1 together with ED-1 in the traumatic penumbra region of the injured brain indicates infiltration of immune cells into the brain due to disruption of BBB.

### GSNO protects BBB integrity by reducing its leakage, enhancing expression of ZO-1 and occludin, and decreasing both edema and the expression of MMP-9

**Figure 2:**
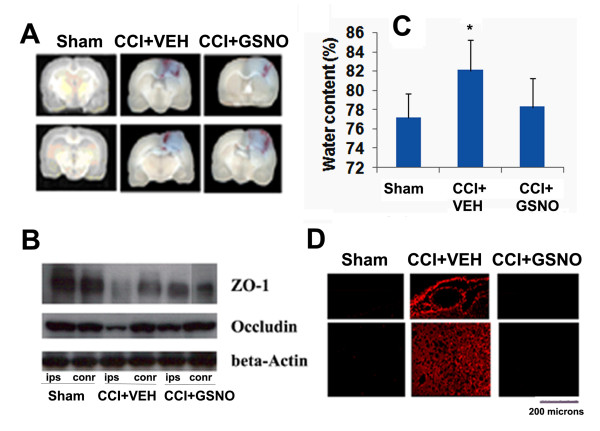
**Effect of GSNO on TBI-mediated BBB disruption, edema and on the expression of ZO-1, occludin and MMP-9 in ipsilateral rat brain after CCI**. Representative photographs showing Evan's blue extravasation at 48 h in 2 out of 6 coronal sections (A). Significant EB leakage was observed in CCI+VEH brain, and the leakage was reduced in the CCI+GSNO group. EB extravasation was not observed in Sham animals (see Table 1). A similar treatment with GSNO decreased the brain water content (edema) in ipsilateral brain (C) measured at 24 h after CCI. Treatment with GSNO also enhanced TBI-mediated reduced expression of ZO-1 and occludin in traumatic penumbral area from ipsilateral hemisphere measures at 24 h following TBI by Western blot (B). TBI-mediated increased expression of MMP-9 was also decreased following GSNO treatment (detected in traumatic penumbral area at 72 h following TBI) (D). Photomicrograph of Evan's blue is the representative of n = 5. Edema data are presented as mean ± SD (n = 5). Western blot and photomicrographs of MMP-9 immunohistochemistry are representative of n = 3 in each group. *p < 0.05 vs. CCI+VEH or Sham. ips, ipsilateral; conr, contralateral.

BBB disruption and edema are the hallmarks of TBI, leading to inflammation, secondary injury, and cell death [30]. An assessment by the EB extravasation method showed reduced BBB leakage in the CCI+GSNO group compared to the CCI+VEH group (Fig 2A). Evaluation of EB color in each animal by an observer blinded to the groups found decreased intensity in the CCI+GSNO group compared to the CCI+VEH group (Table 1), indicating the efficacy of GSNO for BBB protection. The results were further supported by decreased edema/water content in the ipsilateral side of the CCI+GSNO group compared to the CCI+VEH group (Fig 2C). To support that GSNO blocks BBB leakage, we determined the expression of proteins (ZO-1 and occludin, Fig 2B) involved in maintaining the integrity of BBB using Western blot and the expression of MMP-9 (immunohistochemistry, Fig 2D) following TBI in the traumatic penumbral area from the ipsilateral hemisphere. The expression of ZO-1 and occludin was decreased in the CCI+VEH brain (Fig 2B), whereas the expression of MMP-9 was increased (Fig 2D). The expression of MMP-9 was also found increased around the vessel in CCI+VEH brain (upper panel Fig 2D). The treatment with GSNO enhanced the expression of ZO-1 and occludin (Fig 2B), and decreased the expression of MMP-9 (Fig 2D), indicating its potential for BBB protection.

**Table 1:** Effect of CCI and protection by GSNO on BBB permeability assessed by macromolecular tracer dye Evan's blue.

**Group**	**# of animal showing staining/total # of animals**			
	**0**	**+**	**++**	**+++**

Sham	5/5	0/5	0/5	0/5

CCI+VEH	0/7	1/7	3/7	3/7

CCI+GSNO	0/7	4/7	2/7	1/7

Qualitative assessment of Evan's blue (EB) dye using a standard procedure of grading as described by Rapoport et al [77] and as given: 0 = no staining; + = mild staining; ++ = moderate staining; +++ = heavy staining. Experimental details of EB extravasation are described in Methods. Animals were treated with GSNO (0.05 mg/kg) at 2 h following CCI. CCI+VEH and Sham animals were treated with the vehicle (PBS). n = 5 (Sham) and n = 7 (CCI+VEH and CCI+GSNO).

### GSNO reduces the expression of proinflammatory mediator iNOS in macrophage/microglia

**Figure 3:**
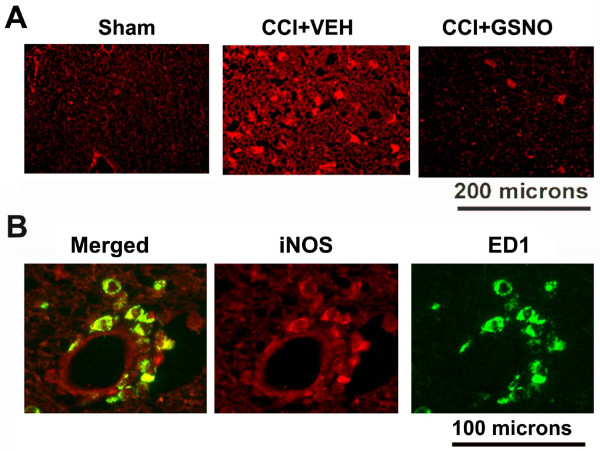
**Effect of GSNO on the expression of iNOS present mainly in macrophages/microglia**. Red fluorescence indicates induced higher expression of iNOS in the traumatic penumbral area of CCI+VEH brain than in the treated (CCI+GSNO) brain determined by immunohistochemistry at 72 h following CCI. Sham animal did not show any significant staining of iNOS (A). Colocalization of the expression of iNOS with ED1 as yellowish fluorescence (B) indicates that iNOS was mainly expressed in macrophages/microglia.

iNOS was highly expressed at 72 h after CCI in the traumatic penumbral area of CCI+VEH compared to Sham brain as shown by immunohistochemical staining. Treatment of the injured animals with GSNO decreased TBI-mediated expression of iNOS (Fig 3A). We also identified that iNOS expression was present mainly in ED1-postive cells (Fig 3B), indicating participation of macrophages/microglia via iNOS in TBI-induced injury.

### GSNO aids recovery and improves neurobehavioral function

**Figure 4:**
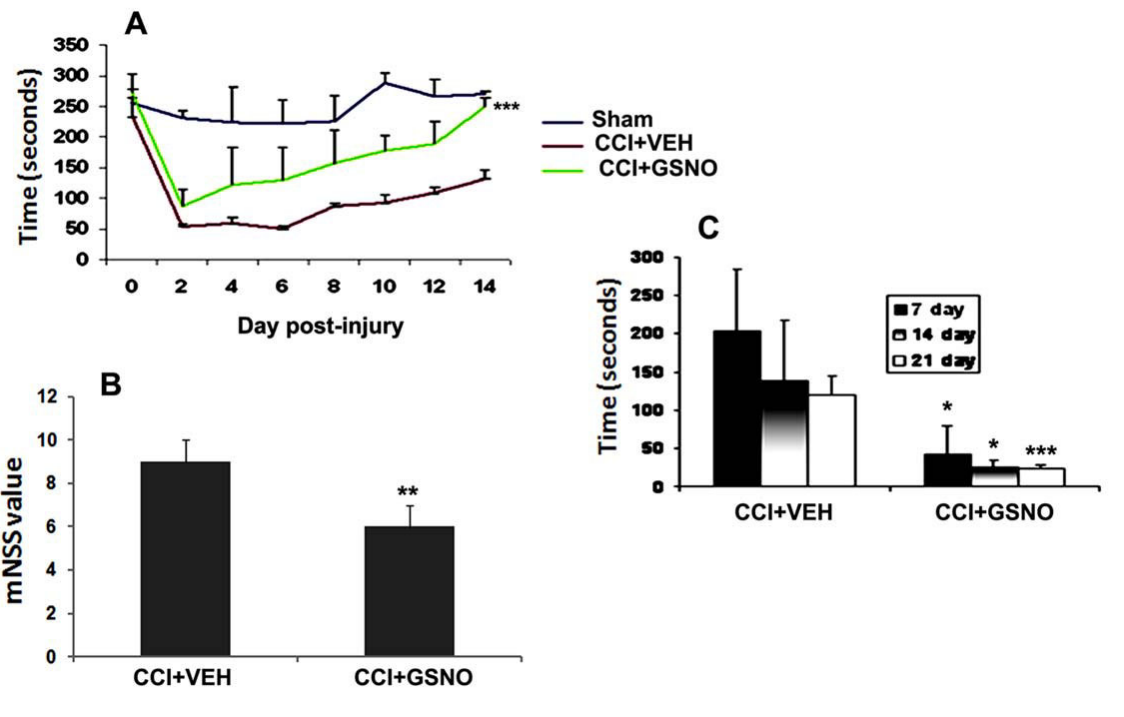
**Effect of GSNO on TBI-mediated deficits in neurobehavioral function**. A treatment at 2 h post-injury following CCI with GSNO improved motor function evaluated by rotarod task (A), decreased modified neurological severity score (mNSS) (B) evaluated on 6^th ^day after CCI and reduced time to remove the stimuli from limbs (tactile strength test) (C). Data are presented as mean ± SD (n = 7). ***p < 0.001, **p < 0.01, *p < 0.05 vs. CCI+VEH.

Neurological deficit was evaluated using three different tests. To assess motor function, we used the rotarod task, which is reportedly the most sensitive vestibulomotor measure [33]. GSNO treatment of the injured animals gradually but significantly improved latency, and hence, motor functions, following TBI (Fig 4A) as evaluated by the rotarod task [50,54]. We also observed improvements in neurological score and sensory testing (Fig 4B-C). Neurological score was determined using the modified neurological severity score test (mNSS) at the 6^th ^day following CCI as described recently in TBI [45]. The score was significantly improved in the CCI+GSNO group compared to the CCI+VEH group (5.8 ± 01.0 CCI+GSNO vs. 9.2 ± 1.0 CCI+VEH, p < 0.01). Sensory dysfunction was assessed using the tactile adhesive removal test [47,48], which was significantly improved in the CCI+GSNO group compared to the CCI+VEH group as shown in Fig 4C.

### GSNO reduces neuronal apoptotic cell death

**Figure 5:**
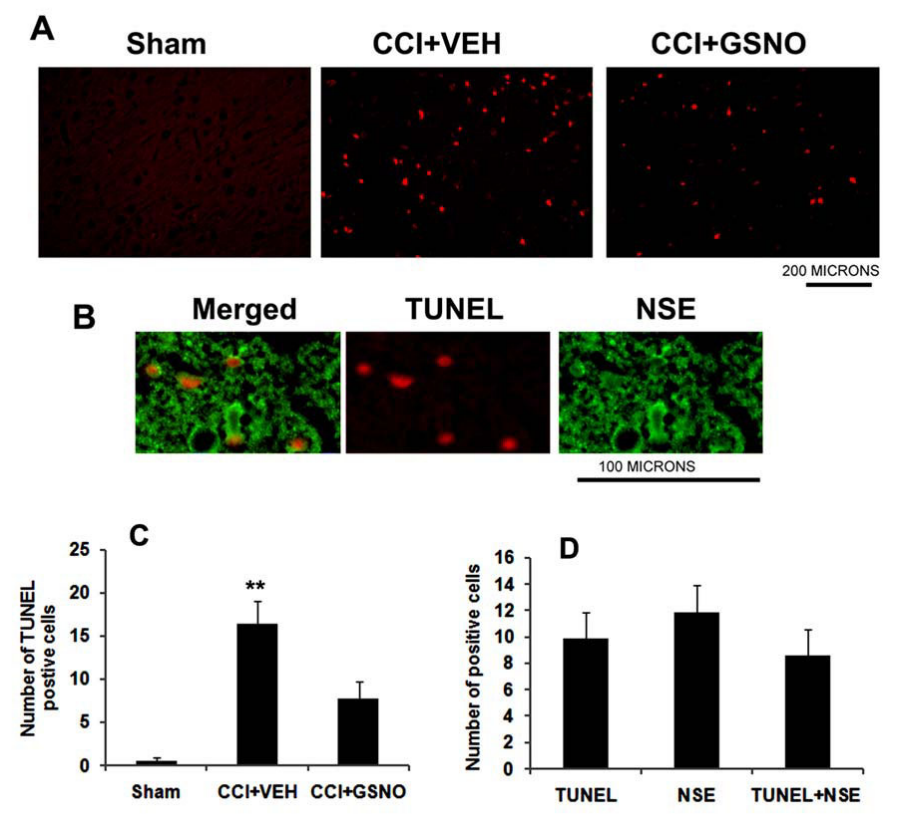
**Effect of GSNO on neuronal apoptotic cell death (TUNEL assay) at 72 h after CCI**. Traumatic penumbral region in CCI+VEH brain had a significantly higher number of TUNEL positive cells compared to CCI+GSNO treated brain. Sham brain had no TUNEL positive cells (A). TUNEL positive cells were found to colocalize with neuronal marker NSE (B). Number of TUNEL positive cells (C) and number of stained positive cells (D) were counted in three different fields and averaged. Data are presented as mean ± SD TUNEL staining was carried out as described in Methods. Photomicrographs are the representative of n = 3 in each group. **p < 0.01 vs. Sham and CCI+GSNO.

DNA fragmentation (measured by TUNEL assay) in the traumatic penumbral area of the CCI+VEH brain at 72 h was increased significantly. GSNO treatment resulted in a decreased number of TUNEL-positive cells (Fig 5A, C). Neither Sham nor the contralateral hemishphere (not shown) of CCI+VEH brain showed TUNEL-positive cells. The fact that TUNEL-positive cells were mainly neurons was confirmed by colocalization of the TUNEL staining with the neuron-specific marker-NSE (Fig 5B, D).

## Discussion

GSNO improved neurobehavioral functions and reduced apoptotic cell death, inflammation and BBB leakage. Other GSNO-mediated effects were decreased contusion volume, improved tissue structure, and reduced edema. GSNO also decreased the expression of MMP-9. In sum, the neurovascular protection rendered by GSNO in the CCI model of TBI is achieved not only through anti-inflammatory and antioxidant mechanisms but also via BBB-protecting activities and mechanisms that aid functional recovery.

Animal models of TBI using the CCI technique are physiologically relevant to TBI in humans. CCI reproduces many of the features of brain injuries, including motor deficits, memory loss, and neuron loss [34,39,55,56]. The severity of injury can be controlled by altering the velocity and depth of the impact and the size of the impactor tip [57]. It is recognized that very severe injury involves several pathways, thus making it difficult to delineate the critical ones. We therefore used a moderate TBI model as described in Methods and reported previously [34,35,39].

Immediately following TBI, the direct trauma and lack of blood flow cause necrotic neuronal death. However, even greater apoptotic neuronal loss occurs hours and days later, caused by secondary injury from cerebral ischemia/hypoxia as well as inflammatory and oxidative stress. Evidence for the existence of a 'traumatic penumbra' (tissue that is most at risk of secondary ischemic injury and that will be most affected by changes in physiology or therapeutic interventions) has been shown in human traumatic head injury [2,3]. In this respect, TBI has similar pathophysiology to ischemic stroke. Both have hypoxia, disrupted BBB, edema, inflammation, neurodegeneration, and neurological deficits. Furthermore, the time-dependant progression of the disease presents a window of opportunity to reduce secondary injury-mediated damage in both stroke and TBI. Regional ischemia early after TBI has been shown to correlate with neurological outcome [2]. The majority of patients who die from TBI demonstrate cerebral ischemia, which may be an important secondary event affecting outcome following TBI. CBF is approximately half normal following TBI in the first 24 h [58], and alterations in vascular functions are mainly due to endothelial dysfunction and reduced NO bioavailability, which leads to oxidative exacerbations and BBB leakage. Decreased levels of NO have been reported in plasma from stroke patients and in animal brain tissue after experimental TBI, indicating an abnormality of NO metabolism after acute injury [59,60]. Decreased NO levels may be responsible for the decrease in CBF and the consequent BBB leakage as well as cell death. To support the pathology associated with the decrease in NO level, NO donors and L-arginine (the substrate of NOSs) are reported to reduce TBI [61,62]. These observations indicate that targeting the NO modulating system is an attractive approach for developing TBI therapy.

To date, major clinical trials with neuroprotective drugs that targeted TBI amelioration have not been efficacious [4]. The failure of therapies targeted only to neuronal protection is, in part, attributable to the lack of concomitant protection of cerebral blood vessels from the secondary injury of inflammation and accumulating oxidative exacerbations. An inhibition of inflammation and reduction of oxidative exacerbations by a post-stroke injury treatment with the NO-modulating agent GSNO resulted in an increased CBF as well as neurovascular protection, leading to improved neurological symptoms in a rat model of experimental stroke [7]. CBF is dependent in part on vasodilation, which is mainly regulated by NO. NO bioavailability in the endothelium is reduced following TBI due to its diffusion-limited reaction with superoxide, resulting in the formation of peroxynitrite. We have reported earlier that GSNO increased CBF in stroke animals [6]; therefore, GSNO possesses the potential to maintain BBB integrity via its hemodynamic activity. Our studies found that NO donors belonging to structurally different classes increase CBF. However, only those NO donors which possessed a nitrosylating property (e.g. GSNO and SNAP) were protective [6], indicating that enhancing CBF only is insufficient to provide neurovascular protection. Therefore, we opted for the nitrosylating agent GSNO to investigate whether it has neurovascular protective effects in experimental TBI similar to that in experimental stroke.

GSNO functions in vivo mainly via nitrosylation of protein thiols in health and disease [63,64]. It is an endogenous nitrosylating agent [8], and its homeostasis is maintained in blood and tissues [65]. Abnormalities in S-nitrosylation cause diseases, as is evident from S-nitrosothiol depletion in asthma [66] and amyotrophic lateral sclerosis [67]. Depletion of plasma-nitrosylated species has also been observed in patients with endothelial dysfunction [68]. However, under certain circumstances, GSNO may release NO and function as an NO donor. Unlike conventional NO donors which release NO quickly and with high density, nitrosylating agents such as GSNO and SNAP release NO slowly and invoke neurovascular protective effects following IR [6,7]. We also reported previously that treatment with GSNO reduces not only neuronal cell death and glial cell inflammation but also reduces endothelial cell activation evaluated as the expression of ICAM-1 and E-selectin [6,7], indicating the potential of GSNO therapy for the protection of BBB following acute brain injury. Later, we found that inhibition of endothelial cell activation was dependent on GSNO-mediated nitrosylation of the p65 subunit of NF-κB [5]. Because activation of endothelial cells via increased expression of ICAM-1 is directly involved in BBB disruption and inactivation of ICAM-1 provides protection in BBB [69], we investigated the GSNO intervention's effect on BBB protection in this study. GSNO decreased EB extravasation (a measure of BBB leakage) following TBI (Fig 2A and Table 1), supporting our *in vitro *results [5] that GSNO protects BBB integrity. We also found decreased expression of ICAM-1 (Fig 1D) and the consequent inhibition of infiltration/activation of macrophages/microglia (ED1) (Fig 1E). Along with the expression of CAMs, the degradation of basal lamina and activation of MMP-9 was also observed following TBI [29]. The observed decreased expression of MMP-9 and protection against degradation of the basal lamina in the GSNO group (Fig 2D) support the efficacy of GSNO for BBB protection. Expression of both ICAM-1 and MMP-9 are regulated by NF-κB; therefore, GSNO may reduce the expression of both MMP-9 and ICAM-1 via inhibition of NF-κB through nitrosylation of the P65 subunit of NF-κB as reported earlier [5]. Overall, GSNO treatment resulted in improved tissue morphology (H&E staining, Fig 1A), reduced contusion volume (Fig 1B), and reduced loss of myelin (Fig 1C), suggesting that these morphometric improvements are achieved due to GSNO-mediated BBB protection. Consistent with our finding of GSNO-mediated BBB protection, the role of GSNO in down regulation of inflammation and protection of the intestinal barrier has also recently been reported [70].

The BBB is a neurovascular system mainly composed of endothelial and astroglial cells with a basal lamina. It is highly discriminatory and has selective permeability inside the brain. Tight junction-associated proteins such as ZO-1 and occludin play critical roles in maintaining BBB functions [71]. We therefore investigated the status of these tight junction proteins and observed that TBI reduced the levels of both ZO-1 and occludin examined at 24 h following CCI (Fig 2B). Protection by GSNO against the TBI-mediated loss of ZO-1 and occludin suggests that GSNO protects endothelial tight junctions and therefore keeps the BBB intact. Interestingly, ZO-1 has been reported to contain a transnitrosylation consensus motif at cysteine amino acid residue 1718 [13]. However, whether 1718 cysteine in ZO-1 is nitrosylated and the consequent effect this nitrosylation would have on BBB integrity are not known.

Traits of the BBB suggest that the effect of a contusion may be disruptive, and disruption of BBB is one of the major causes of secondary injury. After BBB compromise, unwanted cells, debris, and water transmigrate across and infiltrate the BBB, which finally leads to edema, a prominent cause of mortality following TBI. In this study, GSNO decreased edema, likely via BBB protection (Fig 2C). Blood leakage into the parenchyma through the compromised BBB contributes further to inflammation, disrupting cellular defense mechanisms. Blood is also a rich source of iron, which catalyzes free radical (hydroxyl radical) formation, causing instant lipid peroxidation-mediated cell death. Lipid peroxidation, which goes hand-in-hand with inflammation in injured brain tissue, was decreased by the treatment of GSNO in a rat model of experimental stroke [6].

Inflammation, an essential component of TBI, is involved not only in BBB disruption and apoptotic cell death but also hinders the recovery of neurobehavioral functions. Inhibition of cytokine production by anti-inflammatory agents, including minocycline and statins, has been shown to reduce TBI [52,72]. Among the inflammatory mediators, iNOS induction and the consequent product peroxynitrite have been implicated in TBI. Peroxynitrite is formed via a diffusion-limited reaction of superoxide and NO, which damages biomolecules by nitrotyrosine formation. As a consequence, the levels and bioavailability of NO are decreased. Reduction of peroxynitrite via peroxynitrite-reducing agents, including tempol, protects against TBI, indicating the deleterious role of peroxynitrite [73]. Expression of iNOS has been found near necrotic and inflammatory areas mainly in neutrophils/macrophages, where it plays a crucial role in secondary brain damage subsequent to TBI in humans [74], and inhibition of iNOS has protected against injury in TBI animal models [73,75]. We observed that GSNO inhibited the expression of iNOS after the injury (Fig 3), indicating that GSNO has potential to protect the brain against iNOS-mediated neurodegeneration in TBI. We have previously also documented the inhibitory effect of GSNO on iNOS induction in the brain following IR and in rat primary astrocytes and microglial cell lines in culture [7]. The inhibition of NF-κB by GSNO indicates that GSNO may exert an iNOS inhibitory effect in an NF-κB dependent pathway [7].

Therefore, the major focus of TBI research should be protection of neurons from apoptotic cell death by reducing the secondary injury of inflammation and oxidative stress. We have previously reported in our stroke study the potent anti-apoptotic action of GSNO, which likely occurs through GSNO-mediated inhibition of inflammation and caspase-3 activity [7]. GSNO has been known to inhibit the activity of caspase-3 via its nitrosylation [76]. In this study, GSNO also reduced neuronal apoptotic cell death (Fig 5), indicating mechanisms in TBI similar to stroke.

Inflammation is an intrinsic modulator of events which cause neurological deficits and cell death following TBI [51,52]. Improvements in motor functions are the significant indications and endpoint result to determine the efficacy of a therapeutic after acute injury. Therefore, we next examined whether our biochemical results were consistent with neurobehavioral recovery. Three different tests were performed at regular intervals (Fig 4) to assess the functional deficit and recovery of functions of the injured (CCI+VEH) and GSNO-treated (CCI+GSNO) rats. Motor functions, as evaluated by rotarod task, started improving from day 3 (Fig 4A), which correlated well with the integrity of BBB (Fig 2A), edema (Fig 2C), inflammation (Fig 3), and neuronal cell death (Fig 5). A gradual but continuous improvement of motor function together with the recovery of sensorimotor functions (Fig 4C) and cognition (Fig 4B) indicate that GSNO-mediated mechanisms promote recovery and enhance repair mechanisms. In sum, the data from neurobehavioral evaluations accord with the protection of BBB and down regulation of events associated with secondary injury, and these data strongly support the efficacy of GSNO for TBI therapy.

## Conclusion

GSNO protects against TBI by reducing the exacerbations in the neurovascular unit which are involved in induction of BBB leakage, cell death, and neurobehavioral deficits in rats following CCI.

## List of abbreviations

BBB: blood brain barrier; CBF: cerebral blood flow; CCI: controlled cortical impact; CNS: central nervous system; EB: Evan's blue; eNOS: endothelial nitric oxide synthase; GSNO: S-nitrosoglutathione; H&E: hematoxylin and eosin; iNOS: inducible nitric oxide synthase; IR: ischemia-reperfusion; LFB: luxol fast blue; MCAO: middle cerebral artery occlusion; mNSS: modified neurological severity score; NF-κB: nuclear factor kappa B; NO: nitric oxide; NOS: nitric oxide synthase; RNS: reactive nitrogen species; ROS: reactive oxygen species; Sham: sham-operated animals; SNAP: S-nitroso-N-acetyl-penicillamine; TBI: traumatic brain injury; TNF: tumor necrosis factor; VEH: vehicle.

## Competing interests

The authors declare that they have no competing interests.

## Authors' contributions

This study is based on an original idea of MK and IS. MK wrote the manuscript. YI, AS, AG, RKD carried out animal and biochemical studies. AKS critically examined histochemical studies and corrected the manuscript. All authors have read and approved the manuscript.

## References

[B1] Greve MW, Zink BJ (2009). Pathophysiology of traumatic brain injury. Mt Sinai J Med.

[B2] Coles JP, Fryer TD, Smielewski P, Chatfield DA, Steiner LA, Johnston AJ, Downey SP, Williams GB, Aigbirhio F, Hutchinson PJ (2004). Incidence and mechanisms of cerebral ischemia in early clinical head injury. J Cereb Blood Flow Metab.

[B3] Coles JP (2004). Regional ischemia after head injury. Curr Opin Crit Care.

[B4] Jain KK (2008). Neuroprotection in traumatic brain injury. Drug Discov Today.

[B5] Prasad R, Giri S, Nath N, Singh I, Singh AK (2007). GSNO attenuates EAE disease by S-nitrosylation-mediated modulation of endothelial-monocyte interactions. Glia.

[B6] Khan M, Jatana M, Elango C, Paintlia AS, Singh AK, Singh I (2006). Cerebrovascular protection by various nitric oxide donors in rats after experimental stroke. Nitric Oxide.

[B7] Khan M, Sekhon B, Giri S, Jatana M, Gilg AG, Ayasolla K, Elango C, Singh AK, Singh I (2005). S-Nitrosoglutathione reduces inflammation and protects brain against focal cerebral ischemia in a rat model of experimental stroke. J Cereb Blood Flow Metab.

[B8] Schrammel A, Gorren AC, Schmidt K, Pfeiffer S, Mayer B (2003). S-nitrosation of glutathione by nitric oxide, peroxynitrite, and (*)NO/O(2)(*-). Free Radic Biol Med.

[B9] Megson IL (2000). Nitric oxide donor drugs. Drugs of the Future.

[B10] Chiueh CC (2002). S-nitrosoglutathione (GSNO) mediates brain response to hypoxia. Pediatr Res.

[B11] Lane P, Hao G, Gross SS (2001). S-nitrosylation is emerging as a specific and fundamental posttranslational protein modification: head-to-head comparison with O-phosphorylation. Sci STKE.

[B12] Gu Z, Kaul M, Yan B, Kridel SJ, Cui J, Strongin A, Smith JW, Liddington RC, Lipton SA (2002). S-nitrosylation of matrix metalloproteinases: signaling pathway to neuronal cell death. Science.

[B13] Stamler JS, Toone EJ, Lipton SA, Sucher NJ (1997). (S)NO signals: translocation, regulation, and a consensus motif. Neuron.

[B14] Choi YB, Tenneti L, Le DA, Ortiz J, Bai G, Chen HS, Lipton SA (2000). Molecular basis of NMDA receptor-coupled ion channel modulation by S-nitrosylation. Nat Neurosci.

[B15] Nakamura T, Lipton SA (2009). According to GOSPEL: filling in the GAP(DH) of NO-mediated neurotoxicity. Neuron.

[B16] Kluge I, Gutteck-Amsler U, Zollinger M, Do KQ (1997). S-Nitrosoglutathione in Rat Cerebellum: Identification and Quantification by Liquid Chromatography-Mass Spectrometry. J Neurochem.

[B17] Rauhala P, Lin AM, Chiueh CC (1998). Neuroprotection by S-nitrosoglutathione of brain dopamine neurons from oxidative stress. Faseb J.

[B18] Zeng H, Spencer NY, Hogg N (2001). Metabolism of S-nitrosoglutathione by endothelial cells. Am J Physiol Heart Circ Physiol.

[B19] Steffen M, Sarkela TM, Gybina AA, Steele TW, Trasseth NJ, Kuehl D, Giulivi C (2001). Metabolism of S-nitrosoglutathione in intact mitochondria. Biochem J.

[B20] Kaposzta Z, Martin JF, Markus HS (2002). Switching off embolization from symptomatic carotid plaque using S-nitrosoglutathione. Circulation.

[B21] Kaposzta Z, Clifton A, Molloy J, Martin JF, Markus HS (2002). S-nitrosoglutathione reduces asymptomatic embolization after carotid angioplasty. Circulation.

[B22] Molloy J, Martin JF, Baskerville PA, Fraser SC, Markus HS (1998). S-nitrosoglutathione reduces the rate of embolization in humans. Circulation.

[B23] Sehba FA, Ding WH, Chereshnev I, Bederson JB (1999). Effects of S-nitrosoglutathione on acute vasoconstriction and glutamate release after subarachnoid hemorrhage. Stroke.

[B24] Kuo YR, Wang FS, Jeng SF, Lutz BS, Huang HC, Yang KD (2004). Nitrosoglutathione improves blood perfusion and flap survival by suppressing iNOS but protecting eNOS expression in the flap vessels after ischemia/reperfusion injury. Surgery.

[B25] Kuo YR, Wang FS, Jeng SF, Huang HC, Wei FC, Yang KD (2004). Nitrosoglutathione modulation of platelet activation and nitric oxide synthase expression in promotion of flap survival after ischemia/reperfusion injury(1). J Surg Res.

[B26] Lloyd E, Somera-Molina K, Van Eldik LJ, Watterson DM, Wainwright MS (2008). Suppression of acute proinflammatory cytokine and chemokine upregulation by post-injury administration of a novel small molecule improves long-term neurologic outcome in a mouse model of traumatic brain injury. J Neuroinflammation.

[B27] Wada K, Chatzipanteli K, Kraydieh S, Busto R, Dietrich WD (1998). Inducible nitric oxide synthase expression after traumatic brain injury and neuroprotection with aminoguanidine treatment in rats. Neurosurgery.

[B28] Knoblach SM, Faden AI (2002). Administration of either anti-intercellular adhesion molecule-1 or a nonspecific control antibody improves recovery after traumatic brain injury in the rat. J Neurotrauma.

[B29] Suehiro E, Fujisawa H, Akimura T, Ishihara H, Kajiwara K, Kato S, Fujii M, Yamashita S, Maekawa T, Suzuki M (2004). Increased matrix metalloproteinase-9 in blood in association with activation of interleukin-6 after traumatic brain injury: influence of hypothermic therapy. J Neurotrauma.

[B30] Barbaccia JJ, Williams JM (2001). The acute management of head injury. Curr Opin Anaesthesiol.

[B31] Gursoy-Ozdemir Y, Can A, Dalkara T (2004). Reperfusion-induced oxidative/nitrative injury to neurovascular unit after focal cerebral ischemia. Stroke.

[B32] Parathath SR, Parathath S, Tsirka SE (2006). Nitric oxide mediates neurodegeneration and breakdown of the blood-brain barrier in tPA-dependent excitotoxic injury in mice. J Cell Sci.

[B33] Hamm RJ (2001). Neurobehavioral assessment of outcome following traumatic brain injury in rats: an evaluation of selected measures. J Neurotrauma.

[B34] Kline AE, Wagner AK, Westergom BP, Malena RR, Zafonte RD, Olsen AS, Sozda CN, Luthra P, Panda M, Cheng JP, Aslam HA (2007). Acute treatment with the 5-HT(1A) receptor agonist 8-OH-DPAT and chronic environmental enrichment confer neurobehavioral benefit after experimental brain trauma. Behav Brain Res.

[B35] Wagner AK, Kline AE, Ren D, Willard LA, Wenger MK, Zafonte RD, Dixon CE (2007). Gender associations with chronic methylphenidate treatment and behavioral performance following experimental traumatic brain injury. Behav Brain Res.

[B36] Wagner AK, Willard LA, Kline AE, Wenger MK, Bolinger BD, Ren D, Zafonte RD, Dixon CE (2004). Evaluation of estrous cycle stage and gender on behavioral outcome after experimental traumatic brain injury. Brain Res.

[B37] Bayir H, Kagan VE, Clark RS, Janesko-Feldman K, Rafikov R, Huang Z, Zhang X, Vagni V, Billiar TR, Kochanek PM (2007). Neuronal NOS-mediated nitration and inactivation of manganese superoxide dismutase in brain after experimental and human brain injury. J Neurochem.

[B38] Cheng JP, Hoffman AN, Zafonte RD, Kline AE (2008). A delayed and chronic treatment regimen with the 5-HT1A receptor agonist 8-OH-DPAT after cortical impact injury facilitates motor recovery and acquisition of spatial learning. Behav Brain Res.

[B39] Hoffman AN, Cheng JP, Zafonte RD, Kline AE (2008). Administration of haloperidol and risperidone after neurobehavioral testing hinders the recovery of traumatic brain injury-induced deficits. Life Sci.

[B40] Pannu R, Christie DK, Barbosa E, Singh I, Singh AK (2007). Post-trauma Lipitor treatment prevents endothelial dysfunction, facilitates neuroprotection, and promotes locomotor recovery following spinal cord injury. J Neurochem.

[B41] Sanchez Mejia RO, Ona VO, Li M, Friedlander RM (2001). Minocycline reduces traumatic brain injury-mediated caspase-1 activation, tissue damage, and neurological dysfunction. Neurosurgery.

[B42] Fink KB, Andrews LJ, Butler WE, Ona VO, Li M, Bogdanov M, Endres M, Khan SQ, Namura S, Stieg PE (1999). Reduction of post-traumatic brain injury and free radical production by inhibition of the caspase-1 cascade. Neuroscience.

[B43] Weissman DE, Stewart C (1988). Experimental drug therapy of peritumoral brain edema. J Neurooncol.

[B44] Nath N, Khan M, Rattan R, Mangalam A, Makkar RS, de Meester C, Bertrand L, Singh I, Chen Y, Viollet B, Giri S (2009). Loss of AMPK exacerbates experimental autoimmune encephalomyelitis disease severity. Biochem Biophys Res Commun.

[B45] Mahmood A, Goussev A, Lu D, Qu C, Xiong Y, Kazmi H, Chopp M (2008). Long-Lasting Benefits after Treatment of Traumatic Brain Injury (TBI) in Rats with Combination Therapy of Marrow Stromal Cells (MSCs) and Simvastatin. J Neurotrauma.

[B46] Li Y, Chen J, Chen XG, Wang L, Gautam SC, Xu YX, Katakowski M, Zhang LJ, Lu M, Janakiraman N, Chopp M (2002). Human marrow stromal cell therapy for stroke in rat: neurotrophins and functional recovery. Neurology.

[B47] Zhao BQ, Wang S, Kim HY, Storrie H, Rosen BR, Mooney DJ, Wang X, Lo EH (2006). Role of matrix metalloproteinases in delayed cortical responses after stroke. Nat Med.

[B48] Hoane MR, Kaufman N, Vitek MP, McKenna SE (2009). COG1410 Improves Cognitive Performance and Reduces Cortical Neuronal Loss in the Traumatically Injured Brain. J Neurotrauma.

[B49] Fox GB, Fan L, Levasseur RA, Faden AI (1998). Sustained sensory/motor and cognitive deficits with neuronal apoptosis following controlled cortical impact brain injury in the mouse. J Neurotrauma.

[B50] Monville C, Torres EM, Dunnett SB (2006). Comparison of incremental and accelerating protocols of the rotarod test for the assessment of motor deficits in the 6-OHDA model. J Neurosci Methods.

[B51] Wang H, Lynch JR, Song P, Yang HJ, Yates RB, Mace B, Warner DS, Guyton JR, Laskowitz DT (2007). Simvastatin and atorvastatin improve behavioral outcome, reduce hippocampal degeneration, and improve cerebral blood flow after experimental traumatic brain injury. Exp Neurol.

[B52] Chen SF, Hung TH, Chen CC, Lin KH, Huang YN, Tsai HC, Wang JY (2007). Lovastatin improves histological and functional outcomes and reduces inflammation after experimental traumatic brain injury. Life Sci.

[B53] Jatana M, Giri S, Ansari MA, Elango C, Singh AK, Singh I, Khan M (2006). Inhibition of NF-kB activation by 5-lipoxygenase inhibitors protects brain against injury in a rat model of focal cerebral ischemia. J Neuroinflammation.

[B54] Piot-Grosjean O, Wahl F, Gobbo O, Stutzmann JM (2001). Assessment of sensorimotor and cognitive deficits induced by a moderate traumatic injury in the right parietal cortex of the rat. Neurobiol Dis.

[B55] Kline AE, Hoffman AN, Cheng JP, Zafonte RD, Massucci JL (2008). Chronic administration of antipsychotics impede behavioral recovery after experimental traumatic brain injury. Neurosci Lett.

[B56] Colicos MA, Dixon CE, Dash PK (1996). Delayed, selective neuronal death following experimental cortical impact injury in rats: possible role in memory deficits. Brain Res.

[B57] Dixon CE, Clifton GL, Lighthall JW, Yaghmai AA, Hayes RL (1991). A controlled cortical impact model of traumatic brain injury in the rat. J Neurosci Methods.

[B58] Botteri M, Bandera E, Minelli C, Latronico N (2008). Cerebral blood flow thresholds for cerebral ischemia in traumatic brain injury. A systematic review. Crit Care Med.

[B59] Tuzgen S, Tanriover N, Uzan M, Tureci E, Tanriverdi T, Gumustas K, Kuday C (2003). Nitric oxide levels in rat cortex, hippocampus, cerebellum, and brainstem after impact acceleration head injury. Neurol Res.

[B60] Rashid PA, Whitehurst A, Lawson N, Bath PM (2003). Plasma nitric oxide (nitrate/nitrite) levels in acute stroke and their relationship with severity and outcome. J Stroke Cerebrovasc Dis.

[B61] Cherian L, Hlatky R, Robertson CS (2004). Nitric oxide in traumatic brain injury. Brain Pathol.

[B62] Avila MA, Sell SL, Kadoi Y, Prough DS, Hellmich HL, Velasco M, Dewitt DS (2008). L-Arginine decreases fluid-percussion injury-induced neuronal nitrotyrosine immunoreactivity in rats. J Cereb Blood Flow Metab.

[B63] Gaston BM, Carver J, Doctor A, Palmer LA (2003). S-nitrosylation signaling in cell biology. Mol Interv.

[B64] Foster MW, McMahon TJ, Stamler JS (2003). S-nitrosylation in health and disease. Trends Mol Med.

[B65] Martinez-Ruiz A, Lamas S (2004). S-nitrosylation: a potential new paradigm in signal transduction. Cardiovasc Res.

[B66] Que LG, Liu L, Yan Y, Whitehead GS, Gavett SH, Schwartz DA, Stamler JS (2005). Protection from experimental asthma by an endogenous bronchodilator. Science.

[B67] Schonhoff CM, Matsuoka M, Tummala H, Johnson MA, Estevez AG, Wu R, Kamaid A, Ricart KC, Hashimoto Y, Gaston B (2006). S-nitrosothiol depletion in amyotrophic lateral sclerosis. Proc Natl Acad Sci USA.

[B68] Heiss C, Lauer T, Dejam A, Kleinbongard P, Hamada S, Rassaf T, Matern S, Feelisch M, Kelm M (2006). Plasma nitroso compounds are decreased in patients with endothelial dysfunction. J Am Coll Cardiol.

[B69] Bowes MP, Zivin JA, Rothlein R (1993). Monoclonal antibody to the ICAM-1 adhesion site reduces neurological damage in a rabbit cerebral embolism stroke model. Exp Neurol.

[B70] Savidge TC, Newman P, Pothoulakis C, Ruhl A, Neunlist M, Bourreille A, Hurst R, Sofroniew MV (2007). Enteric glia regulate intestinal barrier function and inflammation via release of S-nitrosoglutathione. Gastroenterology.

[B71] Salama NN, Eddington ND, Fasano A (2006). Tight junction modulation and its relationship to drug delivery. Adv Drug Deliv Rev.

[B72] Homsi S, Federico F, Croci N, Palmier B, Plotkine M, Marchand-Leroux C, Jafarian-Tehrani M (2009). Minocycline effects on cerebral edema: Relations with inflammatory and oxidative stress markers following traumatic brain injury in mice. Brain Res.

[B73] Singh IN, Sullivan PG, Hall ED (2007). Peroxynitrite-mediated oxidative damage to brain mitochondria: Protective effects of peroxynitrite scavengers. J Neurosci Res.

[B74] Orihara Y, Ikematsu K, Tsuda R, Nakasono I (2001). Induction of nitric oxide synthase by traumatic brain injury. Forensic Sci Int.

[B75] Clark RS, Kochanek PM, Schwarz MA, Schiding JK, Turner DS, Chen M, Carlos TM, Watkins SC (1996). Inducible nitric oxide synthase expression in cerebrovascular smooth muscle and neutrophils after traumatic brain injury in immature rats. Pediatr Res.

[B76] Mohr S, Zech B, Lapetina EG, Brune B (1997). Inhibition of caspase-3 by S-nitrosation and oxidation caused by nitric oxide. Biochem Biophys Res Commun.

[B77] Rapoport SI, Fredericks WR, Ohno K, Pettigrew KD (1980). Quantitative aspects of reversible osmotic opening of the blood-brain barrier. Am J Physiol.

